# CHIRP-Seq: FOXP2 transcriptional targets in zebra finch brain include numerous speech and language-related genes

**DOI:** 10.1186/s12868-025-00948-6

**Published:** 2025-04-25

**Authors:** Gregory L. Gedman, Todd H. Kimball, Lee L. Atkinson, Daniella Factor, Gabriela Vojtova, Madza Farias-Virgens, Timothy F. Wright, Stephanie A. White

**Affiliations:** 1 Department of Integrative Biology & Physiology, University of California Los Angeles, Los Angeles, CA 90095 USA; 2 Interdepartmental Program in Molecular, Cellular and Integrative Physiology, University of California Los Angeles, Los Angeles, CA 90095 USA; 3 Interdepartmental Program in Neuroscience, University of California Los Angeles, Los Angeles, CA 90095 USA; 4 Department of Biology, New Mexico State University, Las Cruces, NM 88003 USA

**Keywords:** ChIP-Seq, Chromatin-immunoprecipitation, FOXP2, Language, Songbird, Speech, Vocal learning, Zebra finch

## Abstract

**Background:**

Vocal learning is a rare, convergent trait that is fundamental to both human speech and birdsong. The Forkhead Box P2 (FOXP2) transcription factor appears necessary for both types of learned signals, as human mutations in FOXP2 result in speech deficits, and disrupting its expression in zebra finches impairs male-specific song learning. In juvenile and adult male finches, striatal FOXP2 mRNA and protein decline acutely within song-dedicated neurons during singing, indicating that its transcriptional targets are also behaviorally regulated. The identities of these targets in songbirds, and whether they differ across sex, development and/or behavioral conditions, are largely unknown.

**Results:**

Here we used chromatin immunoprecipitation followed by sequencing (ChIP-Seq) to identify genomic sites bound by FOXP2 in male and female, juvenile and adult, and singing and non-singing birds. Our results suggest robust FOXP2 binding concentrated in putative promoter regions of genes. The number of genes likely to be bound by FOXP2 varied across conditions, suggesting specialized roles of the candidate targets related to sex, age, and behavioral state. We interrogated these binding targets both bioinformatically, with comparisons to previous studies, and biochemically, with immunohistochemistry using an antibody for a putative target gene. Gene ontology analyses revealed enrichment for human speech- and language-related functions in males only, consistent with the sexual dimorphism of song learning in this species. Fewer such targets were found in juveniles relative to adults, suggesting an expansion of this regulatory network with maturation. The fewest speech-related targets were found in the singing condition, consistent with the well-documented singing-driven down-regulation of FOXP2 in the songbird striatum.

**Conclusions:**

Overall, these data provide an initial catalog of the regulatory landscape of FOXP2 in an avian vocal learner, offering dozens of target genes for future study and providing insight into the molecular underpinnings of vocal learning.

**Supplementary Information:**

The online version contains supplementary material available at 10.1186/s12868-025-00948-6.

## Background

Vocal learning is a complex phenotype in which organisms learn to accurately imitate sounds and use them in the appropriate social contexts. This trait is a remarkable example of convergent evolution across several distinct avian and mammalian taxa [1] and forms the basis for song and speech learning in songbirds and humans, respectively. Both processes involve developmental critical periods whereby learning is most robust in juveniles, with ongoing maintenance of the learned vocalizations in adulthood [2]. A growing body of work suggests that this phenotypic convergence extends to the level of neural architecture [3] and molecular specialization [4, 5], with analogous motor circuits and gene expression patterns supporting vocal learning across taxa. However, our understanding of the genetic mechanisms that establish the specialized circuitry and expression profiles that underlie vocal learning remains incomplete.

An important candidate gene for accurate vocal learning is Forkhead Box P2 (FOXP2). FOXP2 is a member of the Forkhead Box family of transcription factors which canonically bind to a 6–12 base-pair sequence, or regulatory element, usually in the promoter region of genes, whereby they alter target gene expression [6] through both activation and repression [7] with potentially opposite regulatory effects for the same target in different species (e.g. [8, 9]). The role of FOXP2 in vocal learning was first identified in the KE family, a human cohort with a point mutation in exon 14 of the gene, resulting in reduced DNA binding and speech deficits [10, 11]. A similar phenotype was later demonstrated in a songbird, the zebra finch (*Taeniopygia guttata*), as FOXP2 knockdown in juveniles impaired their ability to learn the songs of their adult tutors [12].

Additional evidence from songbirds highlights the dynamic nature of FOXP2 expression in the brain. Work from Teramitsu and White [13] first described the behavioral regulation of FOXP2 with decreased mRNA and protein levels observed in Area X [14], the vocal-dedicated brain region of the songbird striatum, 2 h after the onset of undirected singing in the morning (a form of vocal practice). This finding has been replicated across multiple studies and species [14–19]. Further, while FOXP2 is highly expressed in the striatum of both sexes of zebra finches, only males engage in vocal learning and only males exhibit singing-linked down-regulation, suggesting a sexually dimorphic distribution of molecular targets within this species. Molecular targets of FOXP2 have been identified in brain tissues from humans [20, 21], the only known primate vocal learner [22], however, the FOXP2 regulatory network in an avian vocal learner, and how it fluctuates across sex, development, and behavior is currently unknown.

Any investigation of transcription factor targets requires a high-quality reference genome assembly, a resource that has only recently become available for the zebra finch [23]. The original zebra finch reference genome was produced using Sanger sequencing which resulted in an accurate but exceedingly fragmented assembly with large sequence gaps [24]. Such gaps often occur in highly repetitive, intergenic regulatory regions, limiting the ability to resolve transcription factor binding sites and subsequent target genes [21]. An updated assembly produced by the Vertebrate Genomes Project using both short-read (Illumina, 10X) and long-read (Pacbio, Bio, Nanopore) technologies has produced a more accurate, contiguous assembly which greatly improves the mappability of next-generation sequencing data [23]. Here, we explore the specific utility of this updated assembly for studying FOXP2 gene regulation.

We used a chromatin-immunoprecipitation and sequencing experiment (ChIP-Seq) to determine the molecular targets of FOXP2 in the zebra finch telencephalon across an informative set of developmental and behavioral conditions, including adult female, adult male, juvenile female, and juvenile male, the latter in both singing and non-singing states. To do so, we developed a bioinformatic pipeline for data analysis using both the original and updated zebra finch reference assemblies. With this pipeline, we quantified a substantial improvement in our ability to identify downstream target genes with the newer assembly. We describe distinct binding profiles in putative promoters across all conditions with distinct gene associations in each condition. Adult males exhibit robust functional enrichment of a set of genes related to speech and language dysfunction in humans that fluctuates as a function of age, sex and behavioral state. This initial evidence of a vocal learning-related regulatory network under the control of FOXP2 in the zebra finch provides dozens of novel gene candidates for further study.

## Methods

### Subjects

All animal use was in accordance with National Institutes of Health and American Veterinary Medical Association guidelines for experiments involving vertebrate animals and approved by the University of California Los Angeles (UCLA) Chancellor’s Institutional Animal Care and Use Committee (ARC-2001-054). Birds were selected from breeding pairs in our zebra finch colony maintained at UCLA. For the ChIP-Seq experiments, we created five treatment conditions, with three unrelated zebra finches per condition: (i) non-singing adult males (> 120d), (ii) non-singing adult females (> 120d), (iii) non-singing juvenile females (65d), (iv) non-singing juvenile males (65d), and (v) singing juvenile males (65d). The comparison between singing and non-singing juvenile males was designed to allow us to examine the behavioral regulation of FOXP2 previously reported by our own and other groups [11–15], and its effects on the number of transcriptional targets identified.

For the juvenile male cohort, young males were housed with their families from hatching, enabling them to form a template of their tutor’s song [25]. At the onset of sensorimotor learning (35d), they were individually housed with a female conspecific until 64d when the female was removed. At 65d, males were selected for either the non-singing (NS) or singing condition (S) using established methods for producing high (NS) or low (S) levels of Area X FOXP2 mRNA and protein [11–15]. The NS condition was enabled by the experimenter sitting near to the bird’s cage in the morning and, if the bird attempted to sing, gently distracting it from singing for 2 h after lights-on. Juvenile males who sang > 10 motifs were not used on that day. Those that sang < 10 motifs in the first 2 h were then left undisturbed for an additional hour prior to sacrifice. This protocol previously resulted in gene expression profiles similar to those of birds that do not sing of their own volition [16], and measurements of corticosterone suggest that it does not induce a stress response in NS birds [14]. To be included in the S group, birds must have spontaneously sung > 90 motifs during the 2 h window. Those that met these criteria were sacrificed 1 h later. As noted, these different levels of singing during the initial 2 h reliably produce birds with either high (NS) or low (S) FOXP2 levels in Area X. The additional 1 h delay after 2 h of singing or non-singing was intended to capture differential FOXP2-mediated transcriptional regulation of target genes [16]. Juvenile males were sacrificed by rapid decapitation and the telencephalon was extracted and flash-frozen on liquid nitrogen.

For the remaining non-singing conditions, adult males, and adult and juvenile females were selected from our aviaries and housed individually the day before use. As with juveniles, these non-singing subjects were monitored for 2 h in the morning (to ensure that males sang < 10 motifs; female zebra finches do not typically sing) and then left undisturbed for an additional hour. All birds were sacrificed by rapid decapitation and the telencephalon was extracted and flash-frozen on liquid nitrogen.

In addition to the ChIP-Seq experiments, three adult males were used for immunohistochemical validation of a putative target gene, calcium/calmodulin-dependent serine protein kinase (CASK). As with the juvenile S and NS conditions, we allowed one individually housed adult male to sing undisturbed in the absence of conspecifics for 2 h in the morning. Another individually housed adult male was monitored and, when necessary, distracted from singing for 2 h. An additional male was housed with a female and the status of his singing over the morning was unmonitored, likely resulting in songs that were directed to the female as well as songs that were not (mixed singing) a condition that can result in intermediate levels of Area X FOXP2 mRNA [13]. Three hours after light onset, males were sacrificed via inhalation overdose (isoflurane), perfused with 4% paraformaldehyde and brains extracted and cryoprotected in sucrose solution. Finally, brains from two additional adult males who were not behaviorally monitored were used for cross-validation of FOXP2 and CASK primary antibodies (Supplementary Figs. 1, 2).

### Chromatin immunoprecipitation

Chromatin immunoprecipitation (ChIP) was performed using ChIP-IT High Sensitivity (Active Motif, Cat. No. 53040) following the manufacturer’s protocol. In brief, brains were minced and crosslinked in a formaldehyde solution. The tissue was disrupted using a hand-held homogenizer for 45 s at 35,000 rpm. A Covaris E220 sonicator was used to sonicate at 25% amplitude, 30 s on, 30 s off, for 30 m to yield 200-1000 bp fragments. A portion of each sonicate was de-crosslinked and quantified by a Nanodrop 1000 (Thermo Scientific, F713). Samples were then split evenly into 2 tubes. A cocktail of 4 μg of each of three anti-FOXP2 primary antibodies was applied to one half (Thermo Fisher, Cat. No. 720031, Abcam, Cat. No. ab1307, and Santa Cruz, Cat. No. sc-517261; see below), while the second was used as an input DNA control. After an overnight incubation, samples were washed, de-crosslinked, and prepped for sequencing.

### Antibody specificity

The three anti-FOXP2 antibodies were designed to target different epitopes of the FOXP2 protein as:

1. Goat polyclonal anti-human FOXP2 antibody from Abcam, Cat. No. ab1307 (Lot No. 1020864-5) raised against amino acids 700 to the C terminus (the exact sequence is indicated as ‘proprietary’), a region with a predicted 100% homology between zebra finch and human FOXP2. This antibody was previously validated for use in detecting zebra finch FOXP2 including by Western blot and peptide blocking [18]; see also [26].
2. Rabbit polyclonal anti-human FOXP2 antibody from Invitrogen Cat. No. 720031 (Lot No. RF236640), raised against a cocktail of peptides, namely human FOXP2 aa 576–587, 670–684, 700–715. These sequences are predicted to be shared between human and zebra finch FOXP2 isoforms.
3. Mouse monoclonal anti-human FOXP2 antibody from Santa Cruz, Cat. No. sc-517261 (Lot No. I1517), raised against amino acids 47–287 of human FOXP2. There is near perfect homology between the human and zebra finch FOXP2 with the exception of two additional Qs in the poly-Q region of the protein in the human isoform.

Double immunofluorescence labeling with the latter two antibodies revealed robust overlap in signals in cerebellar Purkinje neurons (Supplementary Fig. 1A). An additional co-stain showed overlapping signals between the first antibody listed above (Goat polyclonal anti-human FOXP2 antibody from Abcam, Cat. No. ab1307), and the rabbit polyclonal anti-human FOXP2 antibody from Invitrogen, Cat. No. 720031 (Supplementary Fig. 1B). Taken together, these data support the interpretation that all three antibodies detect endogenous zebra finch FOXP2.

To detect CASK protein, a rabbit anti-human CASK antibody from Invitrogen (PA5-96141) was used. The immunogen is the first 300 amino acids of human CASK (NP_0011195261). This sequence is 100% conserved in the predicted zebra finch CASK protein (e.g. XP_030138806.1). This anti-CASK antibody was used in dual immunostains together with the mouse monoclonal anti-human FOXP2 antibody from Santa Cruz (Cat. No. sc-517261). The signals overlapped in cerebellar Purkinje neuronal nuclei and striatal Area X neurons (Supplementary Fig. 2). Neither CASK nor FOXP2 signals were observed in the negative control in which the primary antibodies were omitted (Supplementary Fig. 2A,B,D,E).

### Library preparation and next-generation sequencing

Input and FOXP2 ChIP samples were quantified (Qubit 1.0 Fluorometer) and diluted to 0.5 ng/ul. Library preparation was performed using NuGen Ovation Ultralow Library System V2-32 (Cat. No. 0344-32) per manufacturer’s instructions. Briefly, ChIP samples were end-repaired and sequence-specific adapters were ligated to each sample. Following ligation, the DNA fragments were magnetic bead purified and PCR amplified with the following conditions: (1) 72 °C 2 m, (2) 95 °C 3 m, (3) 98 °C 20 s, (4) 65 °C 30 s, (5) 72 °C 30 s, repeat (3–5) for 15 cycles. The amplified DNA was subjected to a final round of bead purification.

Library preparations were quality-assessed by the Agilent 4200 TapeStation system (Cat. No. G2991AA) using D1000 Screen Tape. The ChIP libraries were quantified by the Qubit 1.0. Fluorometer and diluted to 10 nM. Libraries from each sample were combined and sequenced across 2 lanes by the UCLA Neurogenomics Core (UNGC; https://www.semel.ucla.edu/ungc) by the Illumina HiSeq 4000 sequencer, generating between 15 and 50 million 65 bp single-end reads per library. Reads were demultiplexed by the UNGC.

### Sequence data analysis

We used a standardized computational pipeline to analyze our ChIP-Seq data. First, quality control for raw sequence reads from all samples was conducted using FastQC (v0.11.9) and all reports were aggregated using MultiQC (v1.11). Reads that passed QC are detailed in Table. S1. All test and control samples were high quality (Phred > 30) with no adapter contamination, so no trimming was necessary. All samples were aligned to the most recent high-quality zebra finch reference genome (bTaeGut1.4.pri; RefSeq Accession: GCF_003957565.2) using Bowtie2 (v2.2.5) with default settings. We then used samtools (v1.3.1; -F 4 -q 30) and sambamba (v0.8.1) to filter out all unmapped and multi-mapped reads, retaining only uniquely mapped reads (q > 30) for the downstream analyses. We used Macs2 (v2.2.7.1) to call peaks in all samples independently, then generated a high confidence peak set containing overlapping peaks at the same genomic loci ± 5 bp on either side in 2 or more replicates. We used ChipseekeR (v1.30.3) to annotate these high confidence peaks to genes in each condition. The final peak x gene association table was used as input for gene ontology analysis using gprofileR (v0.2.1, *hsapiens* background) and network analysis using StringDB (v10.0, confidence coefficient = 0.4). Putative promoters were defined as the regions 1000 bp before and after the transcription start site.

### Genome assembly comparison

We compared the number of uniquely mapped, multi-mapped, and unmapped reads from all samples across two reference assemblies and tested for differences in these distributions using two sample T tests implemented in R v4.2.2. The small size of each experimental group (n = 3) precluded formal high-powered statistical tests between conditions, resulting in primarily descriptive assessments of differences observed between age, sex and singing status.

### Gene set enrichment analysis

Genes associated with FOXP2 ChIP-Seq peaks were evaluated from previous studies, including [20, 21, 27]. To assess the degree of shared overlap between these studies, we conducted a gene set enrichment analysis using a hypergeometric test. The density of the hypergeometric distribution for each comparison was calculated using the “phyper” function in R with the parameters *q*, *m*, *n*, and *k* where:

*q* = number of genes with FOXP2 peaks in both our dataset and a previous test study

*m* = number of genes associated with FOXP2 peaks in the previous test study

*n* = number of genes *not* associated with FOXP2 peaks in the previous test study

*k* = number of genes associated with FOXP2 peaks in our study

### Immunohistochemistry

The brains of the adult non-singing male (NS), singing male (S) and unmonitored male were cryo-sectioned in the coronal plane at 15 μm and thaw-mounted onto slides (Superfrost, Fisher Scientific), then stored at -80C until use. Immunohistochemical experiments were conducted simultaneously for dual antigens as previously described [14] using primary antibodies against FOXP2 (1:500 dilution of Santa Cruz Biotechnology SC-517261) and CASK (1:200 dilution of Invitrogen PA5-96141). Signals were visualized using fluorescence-tagged secondary antibodies (1:1,000 dilutions of Alexafluor 488 nm A31620 to detect FOXP2 and Alexafluor 596 A11035 to detect CASK). Coverslips were mounted using ProLong Gold Antifade Mountant with DAPI (360 nm, Molecular Probes, Eugene OR). Images were captured using an AxioImager fluorescent microscope (Carle Zeiss, Thornwood NY), Basler camera and Pylon viewer software (Basler Inc., Exton, PA). Fiji (ImageJ) was used to colorize the images via LUT editor. Background noise was depleted using the contrast editor and a 600 × 600 pixel images was cropped from the original 1920-1200 pixels in a location that most clearly showed cells bodies that express CASK (due to the high background level of this protein in tissues). An air bubble stain in the Nissl image in panel A was retouched to improve clarity.

## Results

### Comparison of reference assemblies

**Fig. 1:**
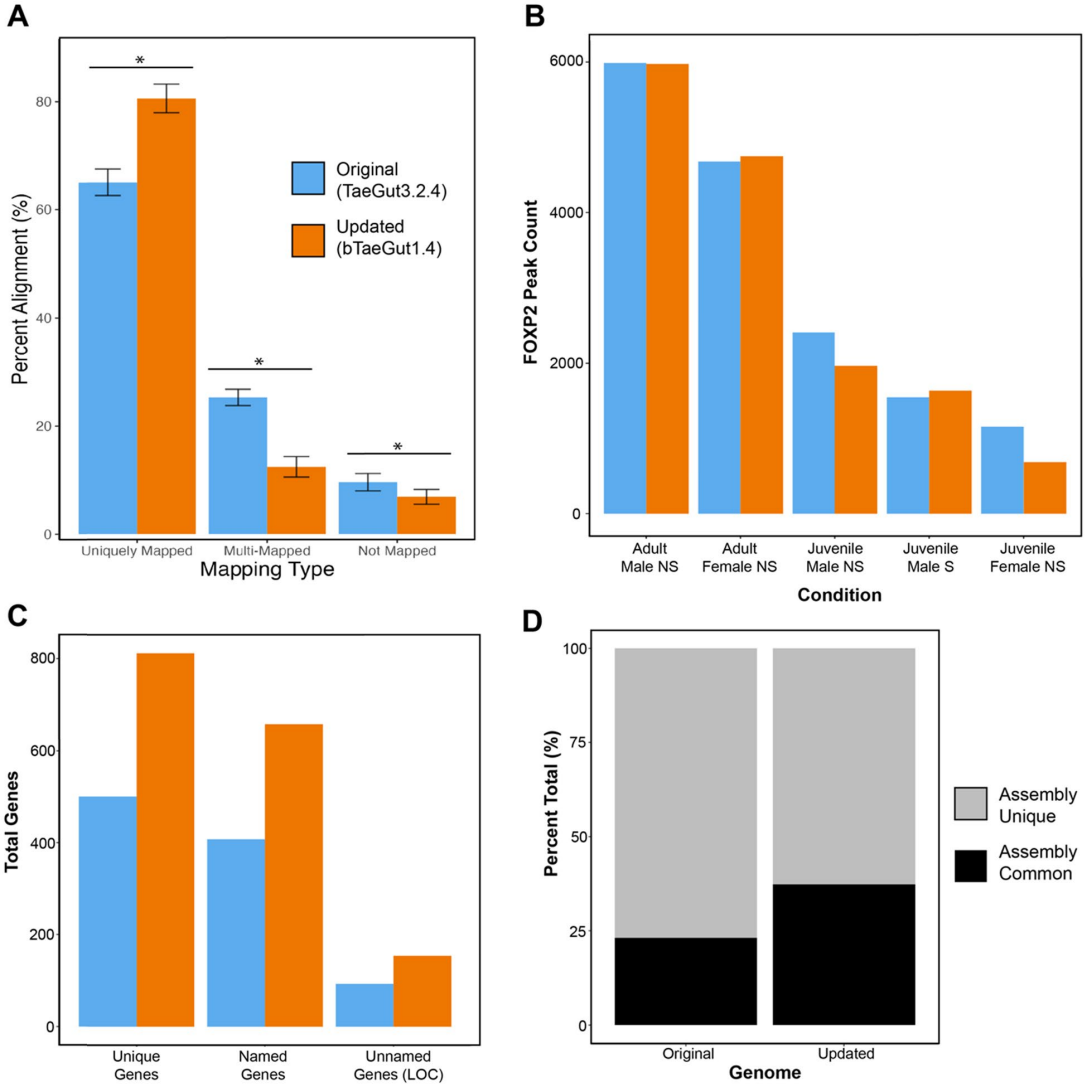
Updated zebra finch reference genome assembly improves sequence alignment and gene assignments. **A** Sequence alignment statistics across all samples using the original (blue) and updated (orange) genomic assemblies. All comparisons were significant at p < 5e-8 or greater. **B** Total number of called FOXP2 peaks by condition using the original and updated genome assemblies. **C** Total number of high-confidence peaks and genes associated with peaks for the adult male condition, comparing the original and updated assemblies. **D** Total percentage of genes associated with peaks that were found using one or both assemblies

We successfully generated 18 million sequence reads on average across the three replicates of each of five conditions (n = 15) that underwent ChIP-Seq using the cocktail of anti-FOXP2 antibodies, and 50 million sequence reads on average in their respective input DNA controls (n = 5; Supplementary Table 1). Reads from the ChIP conditions are enriched for sequences bound by FOXP, while the input control samples contain sequences from all over the genome and include general open chromatin. The libraries showed robust complexity, with a non-redundant read fraction (i.e. proportion of uniquely mapped reads) of 72–87% across all samples (Supplementary Table 1). To determine which reference genome used these data most effectively, we analyzed our data using both the “original” (RefSeq: GCF_000151805.1) and “updated” (Refseq: GCF_003957565.2) zebra finch reference assemblies (Fig. 1). We found that the updated assembly significantly increased unique mapping of sequence reads by ~ 15% (t = 21.41, n = 20, p < 2e-16), and significantly decreased both multiple (t = 26.54, n = 20, p < 2e-16) and unmapped (t = 6.44, n = 20, p = 5.9e-08) reads (Fig. 1A). Surprisingly, we did not find a significant difference between the assemblies in the total number of peaks called from aligned reads, but we did find substantial improvement with the updated assembly when annotating these peaks to genes. For example, in the adult male non-singing condition, comparable numbers of peaks were called using both assemblies (Fig. 1B). When using the original zebra finch reference assembly, we assigned peaks to 500 unique genes, while using the updated zebra finch reference assembly, we assigned peaks to 812 unique genes (Fig. 1C). These increases are not simply due to the annotation of more genes in incomplete regions, as the concordance rate between these gene sets was only 23% (Fig. 1D). These data suggest that the updated zebra finch reference assembly allowed us to more confidently call peaks and assign them to genes. We thus use this updated assembly (bTaeGut1.4 pri) for the remainder of the study.

### Localization of putative FOXP2 binding sites

**Fig. 2:**
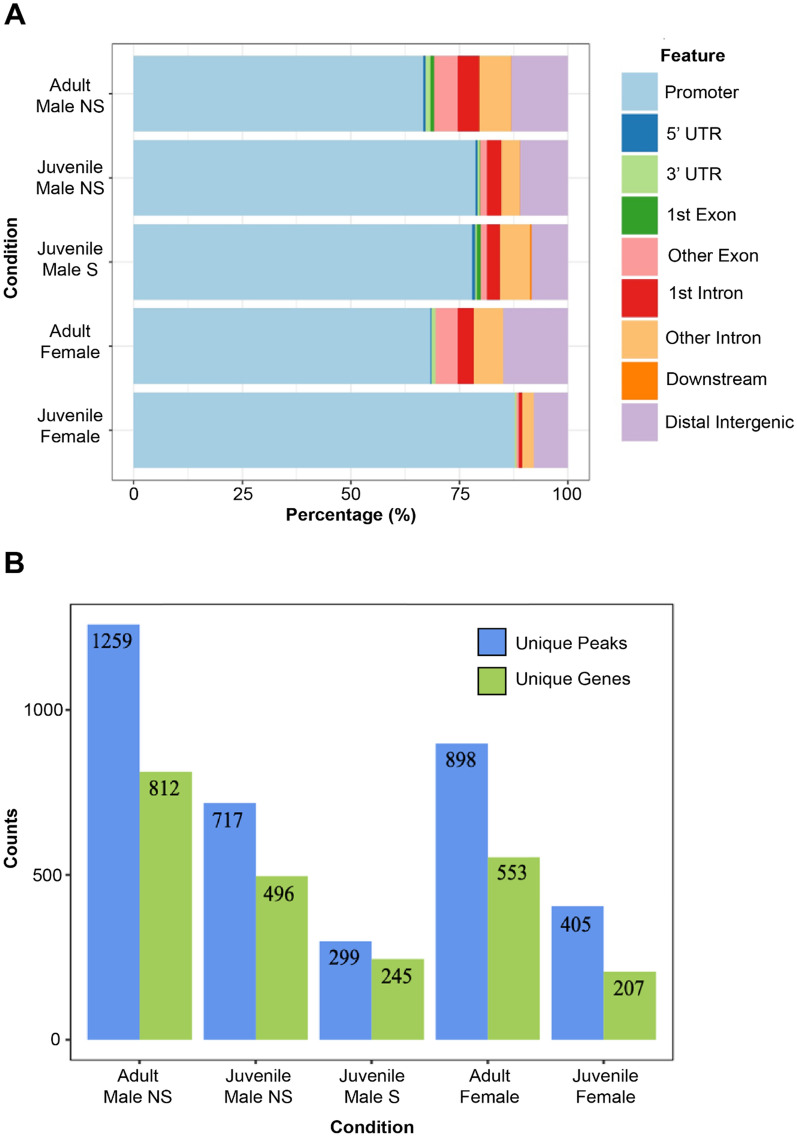
FOXP2 putative promoter binding and gene regulation varies across conditions. **A** Feature plot of called peaks for all conditions. Total peaks for each condition are displayed as a proportion of each annotated feature. **B** Total number of unique peaks (blue) and associated unique genes (green) for each condition in the experiment. *NS* Non-singer, *S* Singer. Promoters were defined as regions 1000 bp before and after the transcription start site

**Fig. 3:**
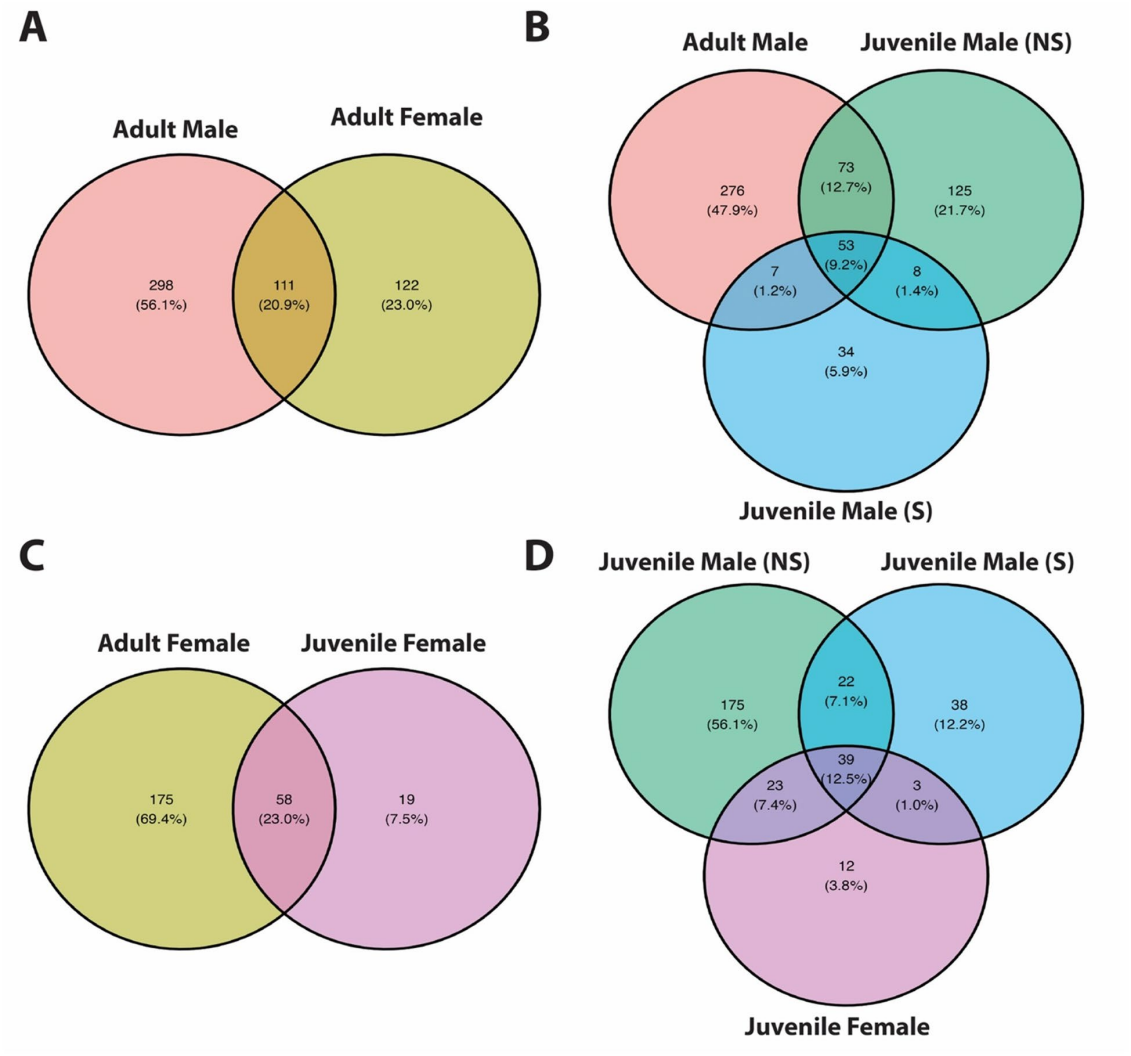
Venn diagrams show the numbers of shared and unshared FOXP2 putative promoter regions of genes among different experimental conditions. **A** Adult conditions: Adult males vs adult females. **B** Male conditions: Adult males vs juvenile non-singing males vs. juvenile singing males. **C** Female conditions: Adult females vs. juvenile females. **D** Juvenile conditions: Juvenile non-singing males vs. juvenile singing males vs. juvenile females *NS* Non-singing, *S* singing. All adult males are non-singing. All gene counts exclude putative promoter peaks within tRNAs

Given FOXP2’s role as a transcription factor, if we successfully isolated and sequenced regions of DNA bound by FOXP2, we would expect an enrichment of peaks within the putative promoter regions of genes when compared to other gene regions, regardless of condition. Indeed, the percentage of peaks that were located within putative gene promoters varied from 63 to 88% across the different conditions, suggesting successful pulldown of FOXP2-bound regions of DNA (Fig. 2A). Although FOXP2 binds primarily in the putative promoter in all conditions, we hypothesized the peak locations and subsequent target genes would vary between conditions given their differences in song learning behavior (e.g. female zebra finches do not learn, young males are engaged in sensorimotor learning or quiescent, and adult males have learned their songs). To test this, we conducted a comparative assessment of all high confidence peaks and their genes (Supplementary Table 2). In general, FOXP2 binding peak frequency increased with age and decreased with singing behavior (Fig. 2B). Both adult male and female zebra finches had markedly more unique peaks associated with unique genes than did juveniles of the same sex, suggesting that more genes are regulated by FOXP2 as the zebra finch matures although the small sample size (three birds per condition) precluded statistical testing. These patterns are illustrated in Venn diagrams representing the number of shared and unshared peaks within putative promoter regions of genes between experimental conditions (Fig. 3).

### Bioinformatic identification of identified FOXP2 gene targets and comparison to prior studies

We bioinformatically identified 812 high confidence FOXP2 gene targets within the adult male zebra finch telencephalon. To assess whether these putative targets are associated with FOXP2, we took multiple approaches, namely comparison of our list with: (i) lists of putative FOXP2 targets previously generated in studies on mammalian nervous tissue [20, 21, 27], (ii) genes previously shown to be differentially expressed in song-dedicated Area X relative to outlying striatum in adult male zebra finches [5], and (iii) genes previously shown to be acutely regulated by singing in Area X [16, 28]. A substantial number of putative targets identified here were cross-validated by one of the above bioinformatic approaches (see below, Tables 1, 2). Only one, CASK, was cross-validated in two separate approaches: This gene was previously identified as a putative FOXP2 gene target in developing mouse brain [27] and as differentially expressed in Area X of adult male zebra finches [5]. For this reason, we selected CASK for an immunohistochemical interrogation to determine whether FOXP2 protein colocalizes with the protein of this putative target within medium spiny neurons of adult male zebra finch Area X (see next sub-section, below).

**Table 1:**
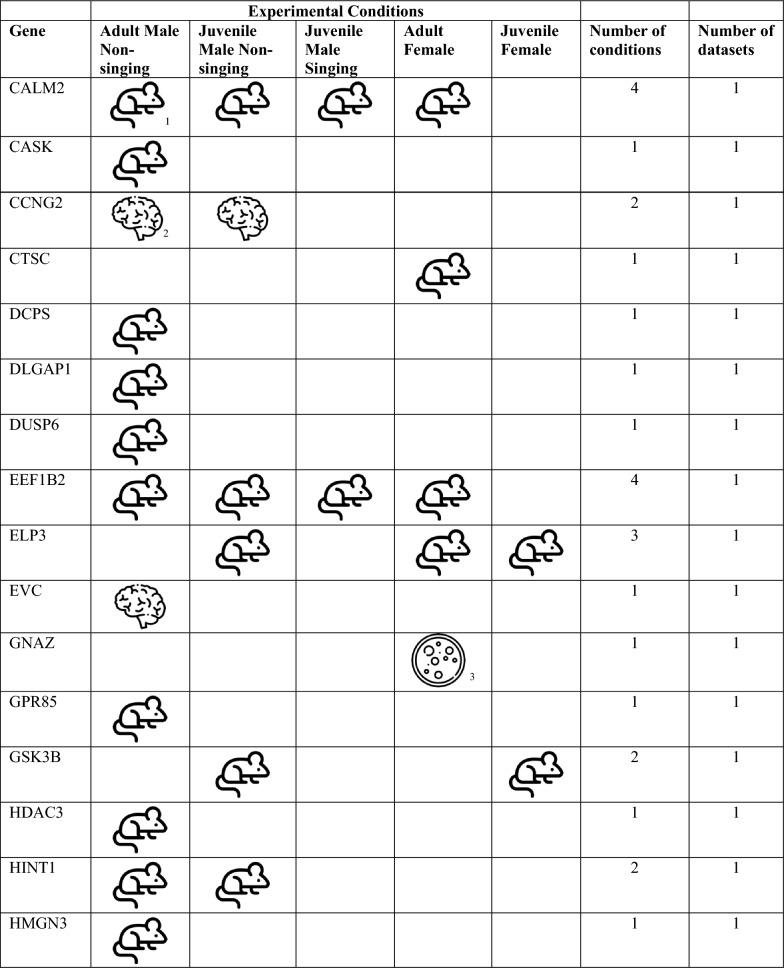

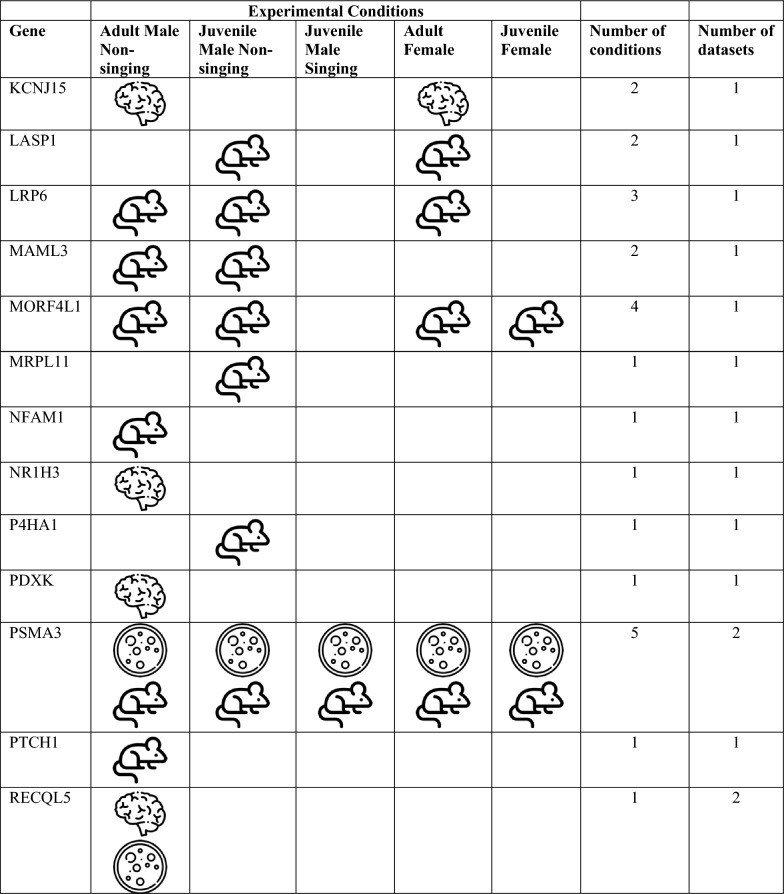

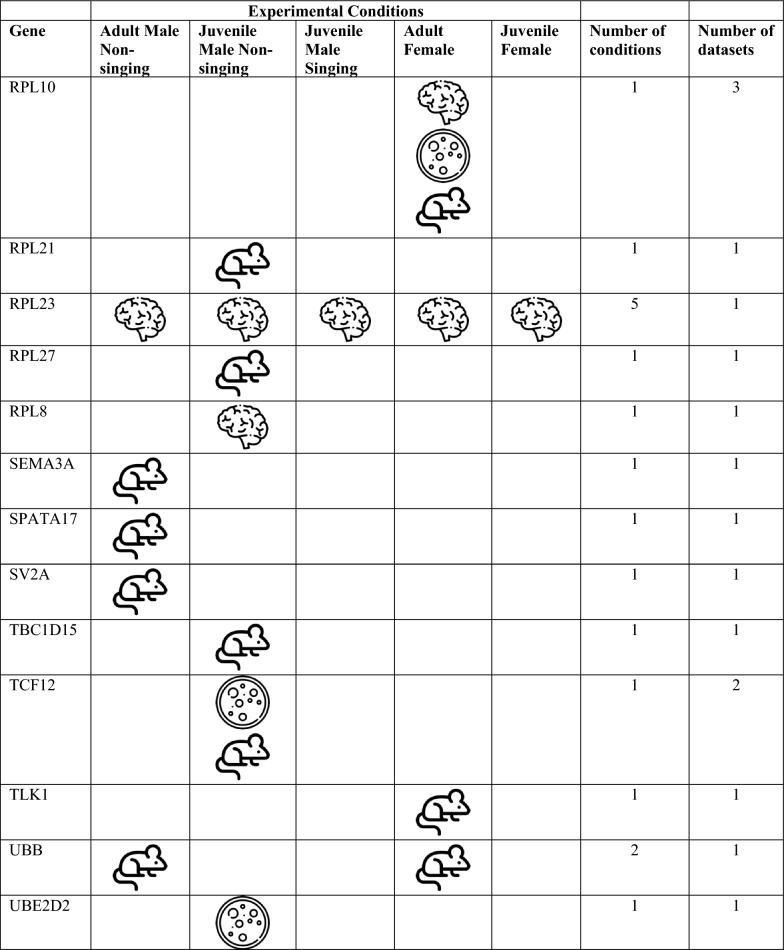

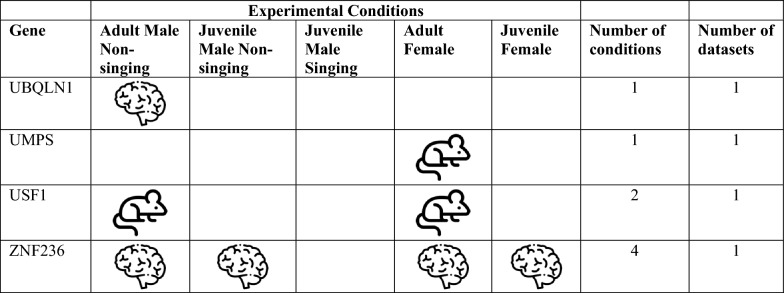
Putative songbird FOXP2 targets shared with mammalian studies

^a^Mouse developing brain [27]

^b^Human developing brain [20]

^c^Human SH-SY5Y cells [21]

**Table 2:** Putative FOXP2 targets in zebra finch with specialized expression in area X^a^, mammalian studies of FOXP2 targets sharing this gene^b,c,d^, and related syndromes in humans

Gene identified as FOXP2 target in this study	Zebra finch chromosome—bTaeGut1.4pri	Mammalian study sharing FOXP2 target	Gene name in mammalian study	Related syndrome	References
AQR	5		PAQR3, PAQR7		
ATP2A2 aka SERCA2	15		ATP1A2, ATP6N1A; ATP51	Parkinson's disease	[30]
CALM1	5		CALM2	Long-QT syndrome	[31]
CASK	1		CASK	Autism spectrum disorder, Prader-Willi syndrome	[32]
FLRT2	5	None		Glass syndrome	[33]
FUS	16		FUSIP1 -	Frontotemporal lobe dementia	[34]
GRINA	2	None			
KIAA0232	4		KIAA0026 KIAA0905 KIAA0979		
MRPL16	5		MRPL11 and 38		
MTMR10	10		MTMR2	15q13.3 microdeletion syndrome	[35]
NFKBIB	34		NFKBIE		
P4HTM	12		P4HA1	HIDEA syndrome	[36]
RALGAPA1	5	None		RALopathies	[37]
RASEF aka RAB45	Z		Multiple RAB isoforms -	Age-related cognitive decline	[38]
SECISBP2L	10	None			
SELENOH	5	None			
SLC31A2	17		Multiple SLC isoforms		
STK24	1	None			
TAF9B	4A		Multiple TAF isoforms		
TUBB3	11	None		TUBB3 E410K syndrome	[39]
WAPL	6	None			

^a^Genes identified as differentially regulated in the zebra finch Area X studies [5, 29]

^b^Mouse developing brain [27]

^c^Human developing brain [20]

^d^Human SH-SY5Y cells [21]

A gene set enrichment analysis against three FOXP2 ChIP-Seq datasets from mammalian nervous tissues [20, 21, 27] revealed 46 genes that exhibited FOXP2 binding in putative promoters in either human or mouse and at least one zebra finch condition (Table 1). While none of these relationships reached the level of significance, this is not surprising, as similar tests that compared putative targets among the three mammalian studies found no significant overlap, with only two genes, CCK and NRN1, being shared among them [20, 21, 27].

Given the importance of FOXP2 expression in Area X of male zebra finches, as a second approach to validation, we examined whether any peaks were located in the putative promoters of genes known to exhibit differential expression in the zebra finch Area X relative to the adjacent non-vocal ventral striatum [5, 29]. We found 21 genes exhibiting either up or down regulation in adult male Area X with at least one FOXP2 binding site in the putative promoter region (Table 2). Of these 21 genes, only one, encoding a protein with unknown function (KIAA0232), was associated with peaks in the juvenile male condition, and only in the non-singer (Table 2). This result suggests that FOXP2 serves distinct regulatory roles in zebra finches across sex, development, and behavior. Of these 21 genes, only one, RASEF (also known as RAB45), is located on the Z chromosome, indicating that chromosomal dosage does not account for most of the regional differences in gene expression. Another, CASK, was previously shown to be a putative FOXP2 target in mice (Tables 1, 2) [27]. An additional 12 of these genes are isoforms or family members of genes previously identified as putative FOXP2 targets in mammalian studies [20, 21, 27]. Moreover, mutations in 10 of these genes are either direct causes of or implicated in nervous system dysfunction including speech and general motor delay (Table 2; see Discussion).

If the genes identified as having peaks are indeed transcriptional targets of FOXP2, then their expression levels are predicted to change as a function of FOXP2 levels. As a third approach to validating these genes as FOXP2 targets, we compared them with a list of transcripts we previously found to be differentially expressed in Area X between singing and non-singing males [16, 28]. In 2012, we employed cDNA microarray technology and weighted gene co-expression network analysis (WGCNA; [40] to assess coordinated changes in gene expression in 26 adults who sang different amounts of song on a given morning. In that study, all 12 of the 60-mer probes for FOXP2 on the microarray indicated decreased FOXP2 expression with greater amounts of singing. Following WGCNA, genes whose expression levels were significantly correlated with singing were grouped into a so-called ‘song-related module’. Any of these previously identified genes are candidates for FOXP2 transcriptional regulation since both FOXP2 and these genes change expression in concert within Area X during an acute 2 h bout of singing.

Overlaps between genes in the adult song-related module [16] and the present study include NTRK2, HOMER1, IRS2, DUSP6 and UBXN2A. NTRK2 encodes the neurotrophin receptor tyrosine kinase 2 which shows one peak of potential FOXP2 binding in the juvenile male singer condition. Previously, Vernes and colleagues [27] identified NTRK2 as a putative target of FOXP2 in the developing mouse brain, providing partial validation for the bioinformatic approach used here.

In addition to adult males, we previously used bulk RNA sequencing to assess singing-driven changes in Area X gene expression of juvenile males [28] at the same age studied here. Among the 5 overlaps mentioned above, HOMER1, which exhibits one peak in the juvenile non-singer condition, was highly correlated with the amount of singing in juveniles in the prior work. Similarly, IRS2 exhibits seven peaks in adult males, two peaks in adult females, and three peaks in the juvenile male non-singing condition and was significantly regulated by singing in juveniles. DUSP6 shows one peak in adult males and was a member of the juvenile song-related module. Moreover, this gene, which encodes dual specificity phosphatase 6, was also shown by Vernes and colleagues to be a putative FOXP2 target [27] providing additional validation of our pipeline. Finally, UBXN2A exhibits one peak in the adult female condition and was significantly regulated by singing in juvenile zebra finch males [28].

### Biological interrogation of an identified FOXP2 target

**Fig. 4:**
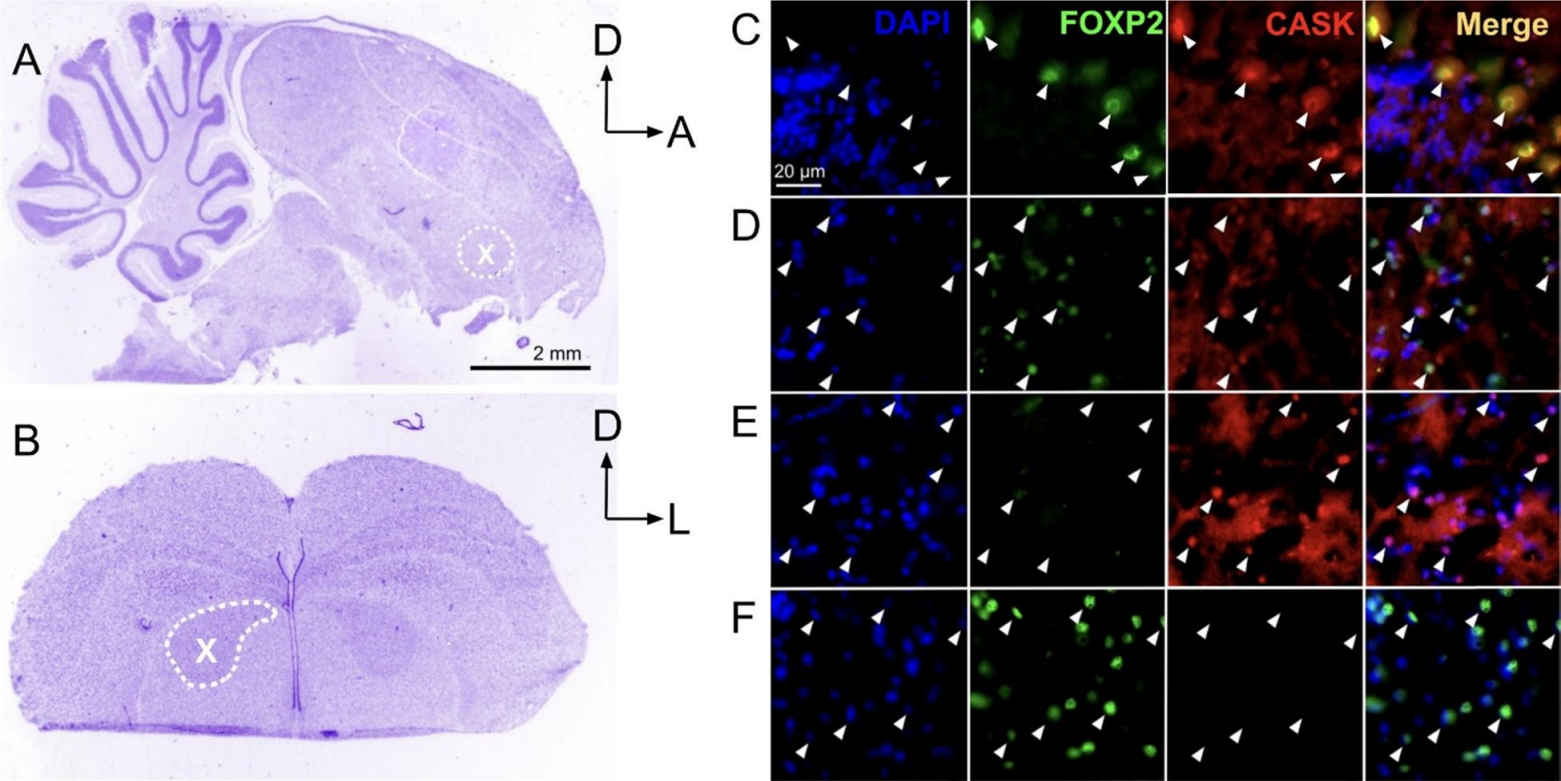
FOXP2 and CASK protein signals co-localize within striatal and cerebellar neurons. **A** Nissl stain of sagittal section of the male zebra finch brain at the level of the cerebellum and Area X (dotted white circle). **B** Nissl stain of the zebra finch telencephalon at the level of striatal Area X (dotted white circle) which is visible bilaterally. **C**–**F** Photomicrographs show immunostain signals for DNA (DAPI-blue), FOXP2 (green) and CASK (red) as well as a merged image (far right panel in each row). **C** As expected, cerebellar Purkinje neurons do not show strong DAPI signals (left panel; white arrowheads) but do co-stain for FOXP2 and CASK. **D** Striatal neurons from an adult male zebra finch housed with a female (mixed singer; see Methods) show co-localization of FOXP2 and CASK signals (white arrowheads). In striking contrast, those from a male who sang alone (**E**) show undetectable FOXP2 signals and strong CASK signals whereas those from a non-singer (**F**) show robust FOXP2 signals and undetectable CASK. See Supplementary Fig. 2 for negative controls. *A* Anterior, *D* Dorsal, *L* lateral. Scale bar = 20um for panels (**C**–**F**)

An immunohistochemical experiment to detect the protein for FOXP2 and that of one of its putative gene targets, CASK, revealed co-localized expression within Area X neurons of an adult male housed with a female (Fig. 4), providing further support that our in silico results have biological relevance. In line with prior work showing singing-driven down-regulation of FOXP2 in Area X, only CASK, and not FOXP2, was detected in Area X neurons of a male who sang by himself for 2 h in the morning. Conversely, only FOXP2, and not CASK was detected in Area X neurons of a male who did not sing. These qualitative findings of inverse expression levels suggest that FOXP2 represses CASK within Area X. Outside of Area X, robust FOXP2 and CASK signals were co-detected in cerebellar Purkinje neurons (Fig. 4).

### Functional processes of putative FOXP2 targets

**Fig. 5:**
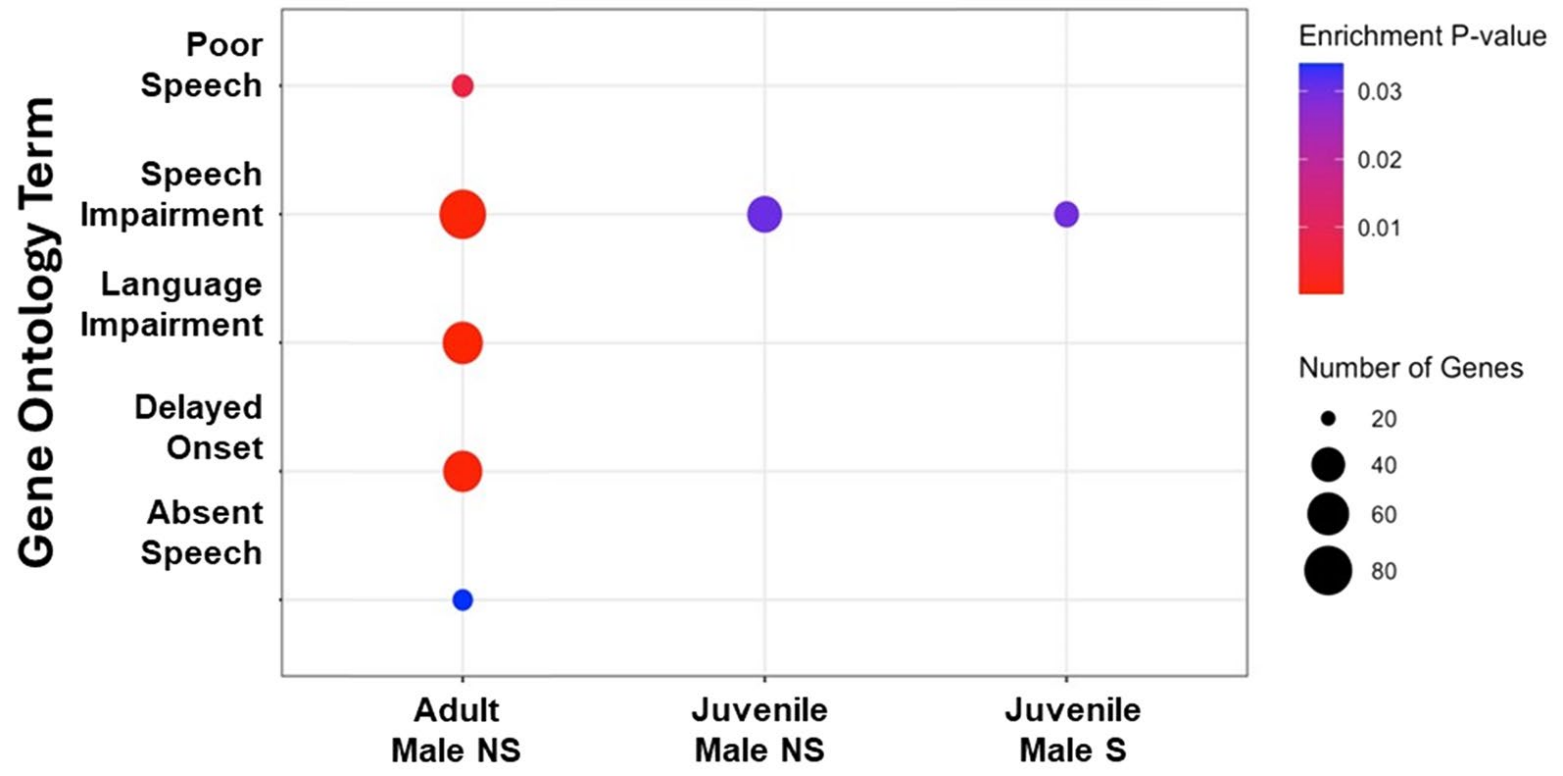
In zebra finch males, but not females, putative FOXP2 targets are implicated in human speech and language. Dot plot highlights speech/language related GO terms from gprofileR. Color of each dot denotes significance after multiple test corrections (Enrichment P-value; FDR < 0.05) and the size of each dot denotes the number of genes found in the given term and condition. The number of genes for each GO term for the three conditions are: Adult Male NS: Poor Speech = 23, Speech Impairment = 78, language impairment = 57, Delayed Onset = 54, Absent Speech = 22, Juvenile Male NS: Speech impairment = 43, Juvenile Male S: Speech Impairment = 26. Adult and juvenile female birds are not displayed as they had no enrichment for the plotted terms. A full list of significant GO terms for each condition can be found in Supplementary Table 2

We next wanted to better understand the overall functional processes of putative FOXP2 target genes identified in each condition. We conducted a gene set enrichment analysis, using the unique set of targets per condition as input (Fig. 2B, Supplementary Table 3). Each condition had hundreds of GO terms (range 775–1733) involved in a wide range of processes including cell signaling, neurogenesis, and axon guidance. Interestingly, we found that FOXP2 targets were enriched for genes related to human speech and language in males only, consistent with the sexually dimorphic vocal learning in this species (Fig. 5). These functions include poor or absent speech, speech and language impairment, and delayed speech onset, all consistent with the phenotypes found in the KE family following FOXP2 mutation [10].

The non-singing adult male zebra finch exhibited the most putative FOXP2 target genes known to be involved in human speech with many fewer found in the non-singing juvenile male (Fig. 5), suggesting the baseline vocal learning regulatory network of FOXP2 expands with development and song learning. The singing juvenile male condition exhibited even fewer target genes related to human speech, consistent with less FOXP2 protein being available to bind to its targets in this behavioral condition [14, 18]. Overall, these data suggest that the FOXP2 transcription factor targets genes involved in human speech and language and that the degree of this regulation depends on sex, age, and singing.

**Fig. 6:**
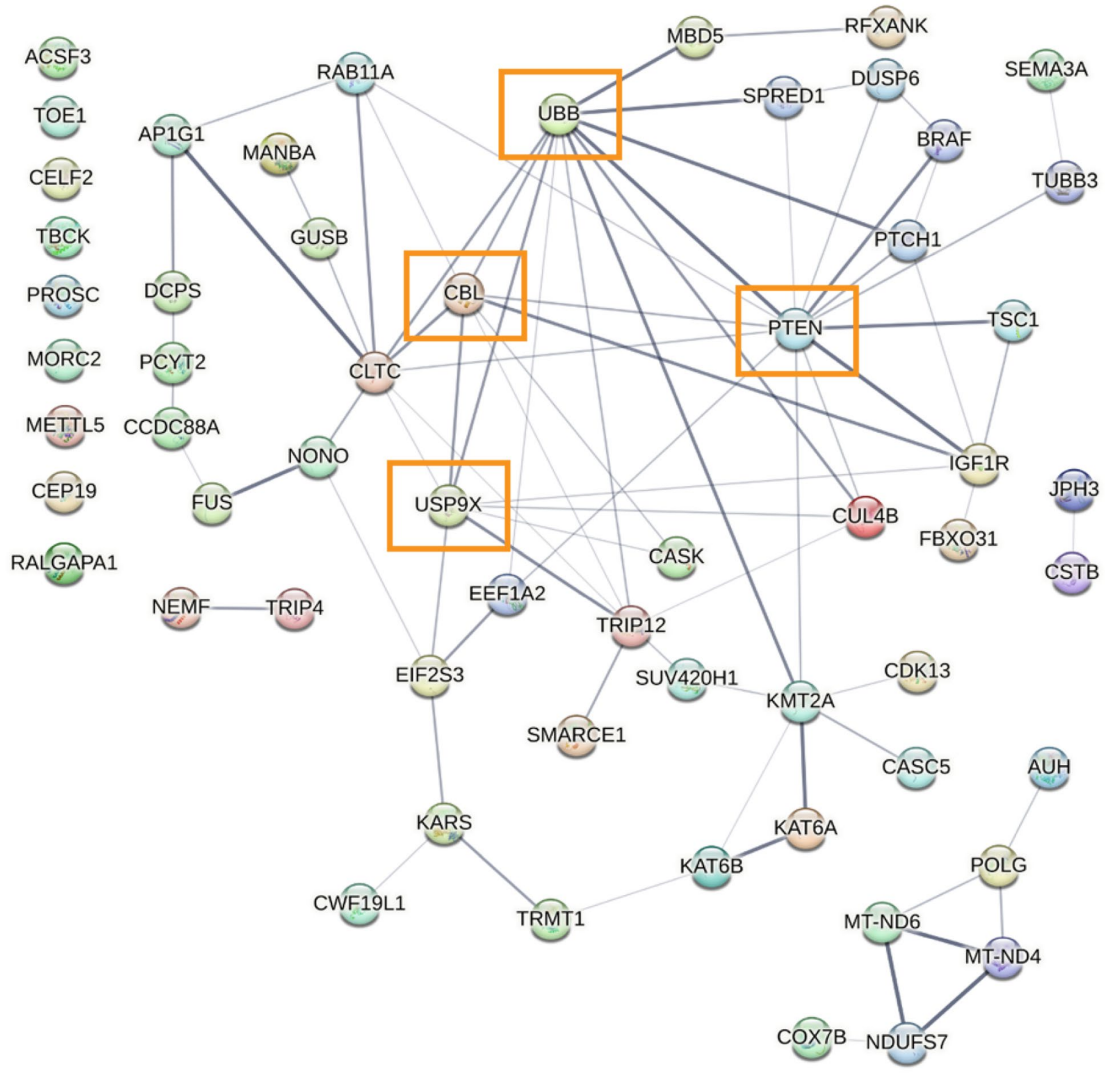
Adult male zebra finch regulatory network highlights molecular targets of FOXP2, many of which are involved in human speech/language dysfunction. Protein–protein interaction network for all genes associated with human speech/language dysfunction in the adult male zebra finch with FOXP2 peaks in their promoters (Fig. 4). Network constructed using StringDB (v10.0). Lines between nodes (genes) denote confidence of interaction with all connections > 40% confidence. All unplaced nodes are clustered on the left of the network. Orange boxes highlight important genes in the ubiquitination pathway

Given the robust enrichment for speech and language-related functions in the FOXP2 candidate target genes in adult male zebra finches, we investigated whether these genes may interact in a functional network. Using StringDB, we defined a protein–protein network from all FOXP2 putative target genes involved in speech and language in the adult male zebra finch telencephalon (Fig. 6). Of the 61 zebra finch genes, 57 were recognized by the network algorithm, and 48 of these genes (84%) formed a functional network with each other. Most notable was UBB, a highly conserved gene coding for ubiquitin which is involved in a number of cellular processes such as protein trafficking and degradation [41]. In the adult male, the UBB promoter contains the most FOXP2 peaks out of any condition, suggesting that strong regulation of free-floating ubiquitin levels is important for maintenance of learned vocalizations in the adult male zebra finch.

## Discussion

In this study, we provide the first detailed description of the putative molecular targets of FOXP2 in the vocal learning zebra finch across sex, development, and behavioral conditions. This analysis was enabled by a new high-quality reference genome assembly, highlighting the importance of robust computational resources for accurate biological conclusions. Improvements to the genomic annotation, including adding previously missed genes, cleaning up spurious exons, and defining upstream promoters [24] have all significantly aided the identification of FOXP2 binding peaks and their association with target genes. Using the new assembly, we found evidence for FOXP2 regulation of ~ 60 genes in adult male zebra finch telencephalon that are involved in human speech and language function, with fewer such genes found in non-singing juveniles and even fewer in juvenile singers, indicating both developmental and behavioral changes in regulation. This dataset highlights a functional network composed of dozens of candidate genes that are targets for further study for their role in vocal learning function in zebra finch and across vocal learning taxa.

The finding of 46 shared putative target genes between our present study on zebra finches and at least one of three prior studies on mammalian nervous tissue, while not statistically significant, is remarkable given that the overlap in target genes among those mammalian studies was limited to two genes, CCK and NRN1 (Table 1) [20, 21, 27]. The lack of commonality among mammalian studies likely reflects the different tissue sources: Spiteri and colleagues [20] examined human fetal basal ganglia and inferior frontal cortex [20] whereas Vernes and colleagues examined human SH-SY5Y cell lines in their 2007 study [21] and reported on whole embryonic mouse brain in 2011 [27]. Nonetheless, those prior studies highlight important consistencies in biological themes, notably neurite outgrowth and synaptic plasticity. All three mammalian studies used microarrays to identify putative targets whereas the present study used DNA sequencing, a methodological difference that reflects on-going technological advances. An even greater number of putative targets identified here are related to isoforms previously identified in prior mammalian work (see below).

Among the 21 putative targets we identified that are also differentially expressed in Area X (Table 2) [5], the RASEF gene (formerly known as RAB45) is the only one located on the Z chromosome. RASEF is a member of the Rab family of GTPases involved in membrane trafficking. In mammals, many RAB isoforms are putative FOXP2 transcriptional targets in the brain tissues [20, 21, 27]. RASEF is part of a novel locus associated with attention deficits identified in a meta-analysis of age-related cognitive decline in 3,045 individuals aged ≥ 65 [38]. Other genes identified in our analysis that are linked with human syndromic brain phenotypes include: TUBB3 (pruning of misguided axons during development [39]; FUS (Fronto-temporal lobe dementia [34]; P4HTM (HIDEA syndrome [36]; and MTMR10 (human 15q13.3 microdeletion syndrome [35]. In mammals, MTMR2 has been identified as a putative FOXP2 target [27] that is associated with neurite outgrowth, providing further support for the bioinformatic pipeline used here.

Only one gene, CASK, was identified as a putative gene target in zebra finches (the present study) and in mice [27], and also exhibited differential expression in zebra finch Area X (Tables 1, 2) [5, 29] We found that signals for CASK protein colocalized with those for FOXP2 within single neurons in zebra finch Purkinje neurons of the cerebellum and in striatal Area X of an unmonitored male housed with a female (Fig. 4D; Supplementary Fig. 2), providing support for their biological interaction. Signal strengths for the two proteins exhibited an inverse relationship (Fig. 4) depending on whether the bird had sung alone (4E) song or had not sung (4F). The CASK gene lies on zebra finch chromosome 1 and encodes a calcium/calmodulin-dependent serine protein kinase anchored to the neuronal membrane at synapses. There, its CaM-kinase domain phosphorylates itself as well as the presynaptic protein neurexin-1 [42, 43]. CASK translocates to the nucleus and interacts with transcription factors to regulate gene expression [32] including that of NECDIN, RLN and the NMDA receptor subunit 2b [44].

In humans, the CASK gene is X-linked and its mutation leads to FG syndrome 4, a form of X-linked mental retardation [32]. Recently, a de novo variant of CASK was found to cause a neurodevelopmental disorder in a 9 year-old boy with severe psychomotor delay [45]. CASK is part of a signaling pathway that includes the widely validated autism susceptibility gene CNTNAP2 and the Prader Willi syndrome gene NECDIN [32]. Zhang and colleagues ([32]; 2023) showed that CNTNAP2 undergoes proteolytic cleavage and its intracellular domain promotes the nuclear translocation of CASK to affect NECDIN expression. Remarkably, viral-driven expression of NECDIN in the Cntnap2^−/−^ mouse model of autism normalized the social deficits of these mice. The authors conclude that the CNTNAP2-CASK-NECDIN signaling pathway plays a critical function in ASD [32].

Our analysis did not identify two genes, VLDLR1 and CNTNAP2, that were previously validated as direct transcriptional targets of FOXP2 in humans and zebra finches [8, 9, 46]. Interestingly, we and others previously identified *VLDLR* mRNA as being regulated by singing in zebra finch Area X [16, 47], and part of a song-related gene module. Our prior work used tissue punches of Area X, whereas, for technical reasons (see below), the present study used the entire telencephalon. Similarly, Adam and colleagues [46] specifically targeted Area X with lentiviral injections to knock down FOXP2 levels, leading to altered CNTNAP2 expression. Here, the inclusion of pallial and striatal tissues outside of Area X likely diminished our ability to detect these associations.

The possibility of a specialized role of FOXP2 in female zebra finches is intriguing given its prominent role in vocal learning in males [12, 48]. We found evidence for female-specific FOXP2 binding in genes associated with ribosomal biogenesis, suggesting differences in protein synthesis between the sexes. Humans [49], mice [50], and yeast [51] exhibit a wide variety of specializations in ribosomal genes across tissues, and *Drosophila* exhibit a sex-specific pattern of ribosomal genes expression in their testes and ovaries [52]. Given the sexual dimorphisms in neural circuitry governing vocal learning in the zebra finch, these female-specific binding events could represent FOXP2 repression of genes that facilitate the synthesis of vocal learning-related proteins in males. Should this be the case, we would not expect to see these sex-specific patterns in songbird species in which females also learn and produce song, or in parrots where call learning occurs in both sexes [53–55].

Many of the putative FOXP2 targets we identified in adult males are genes involved in the cellular ubiquitination pathway and are critical nodes in the speech/language regulatory network, including UBB, USP9X, and CBL. Ubiquitination also influences PTEN function, another gene in this network with mutations associated with communication deficits in autism spectrum disorders [56]. However, the directionality of regulation of these target genes is currently unknown. FOXP2 is canonically thought to serve a repressive role in gene regulation [7], and strong repression of free-floating ubiquitin (UBB), as well as ubiquitin ligases (CBL) and proteases (USP9X), could serve to maintain the current ubiquitin profile in the brain. One way to test this idea would be to repeat these experiments using a singing adult male condition, with the hypothesis that the peaks indicating FOXP2 binding around these genes would disappear, leading to disinhibition and providing flexibility to the ubiquitination state of the brain.

A limitation of this study is that data were obtained from whole telencephalic lysates, rather than solely from song control regions. The reason for this was technical, as limitations in cell number at the onset of these experiments precluded the use of such a small brain region from individual birds, while potential inter-individual variability made pooling individuals to increase cell number undesirable. However, it is likely that Area X provides the primary source of behavioral regulation in our signal, as previous studies have not observed variation in *FOXP2* levels in other telencephalic regions as a function of singing [13, 15]. In addition, using RNA-Seq data from an adult non-singing zebra finch Area X and surrounding striatum [5], we found that several of the FOXP2 target genes from the matching condition in this study exhibit differential expression (Table 2 and Supplementary Table 4). Additional experiments using RNA-seq and ATAC-seq [57] to profile transcriptomic activity in Area X of juvenile males before and after singing, as well as developing female zebra finches, are necessary to determine the extent of FOXP2 regulation in this region.

Overall, this work advances our understanding of the molecular mechanisms underlying the rare trait of vocal learning. Since a role for FOXP2 in human speech and language was first established [10], molecular pathways governed by FOXP2 in human tissue have been identified [20, 21, 27, 58, 59], leading to the hypothesis of similar patterns of regulation in other vocal learning species such as songbirds. The present work provides support for the hypothesis of convergence of FOXP2 transcriptional networks across vocal learning songbirds, humans and potentially with other lineages that exhibit vocal learning. Such similarity would suggest shared constraints on the evolution of this complex trait and provide insights to rescuing deficits in these molecular pathways in the future.

## Supplementary Information

Supplementary material 1.

Supplementary material 2.

Supplementary material 3.

Supplementary material 4.

Supplementary material 5.

Supplementary material 6.

## Data Availability

The BioProject accession link for this study is https://www.ncbi.nlm.nih.gov/sra/PRJNA1114379.
