# Supplementary material for: CHIRP-Seq: FOXP2 transcriptional targets in zebra finch brain include numerous speech and language-related genes
Source: BMC Neurosci. 2025 Apr 25;26:29. doi: 10.1186/s12868-025-00948-6 (PMC12032786; doi:10.1186/s12868-025-00948-6)
Supplement: Supplementary file 1 — Supplementary material 1. [file 12868_2025_948_MOESM1_ESM.docx]

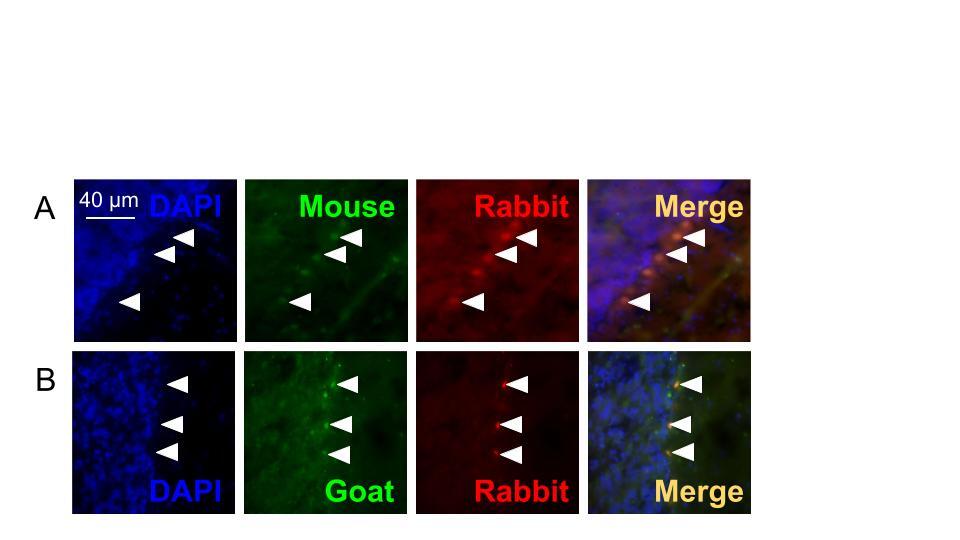
**Supplementary Figure 1. Co-localization of signals from three FOXP2 antibodies used in ChIP-seq.** Photomicrographs show immunostain signals for DNA (DAPI-blue) and FOXP2 (green/red) as well as a merged image (far right panel in each row). Co-localization is indicated by white arrows as well as yellow puncta in merged images. **A)** Dual immunostain against FOXP2 using mouse anti-FOXP2 (green; Santa Cruz Biotechnology, Cat. No. sc-517261) and rabbit anti-FOXP2 (red; Invitrogen, Cat. No. 720031) antibodies. **B)** Dual immunestain against FOXP2 using goat anti-FOXP2 (green, Abcam, Cat. No. ab1307) and rabbit anti-FOXP2 (red) antibodies. As expected, cerebellar Purkinje cells do not show strong DAPI signals.
