# Supplementary material for: CHIRP-Seq: FOXP2 transcriptional targets in zebra finch brain include numerous speech and language-related genes
Source: BMC Neurosci. 2025 Apr 25;26:29. doi: 10.1186/s12868-025-00948-6 (PMC12032786; doi:10.1186/s12868-025-00948-6)
Supplement: Supplementary file 2 — Supplementary material 2. [file 12868_2025_948_MOESM2_ESM.docx]

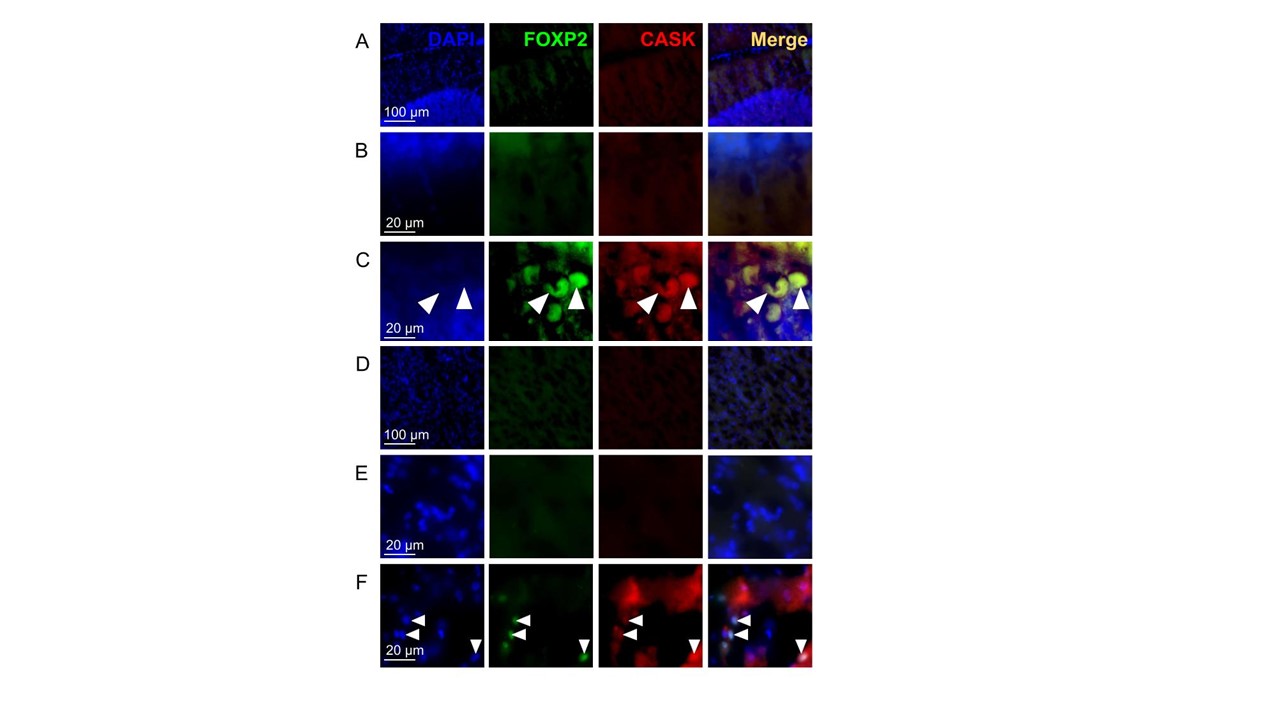
**Supplemental Figure 2. Dual immunofluorescent labeling of CASK and FOXP2 signals in the cerebellum and striatum. (A)** Low power and **(B)** high power images of no-primary antibody controls in zebra finch cerebellum for comparison to **(C).** Arrowheads point to Purkinje neurons indicated by the lack of DAPI staining and show overlap in CASK and FOXP2 signals**.** **(D)** Low power and **(E)** high power images of no primary antibody controls in zebra finch striatal Area X for comparison to **(F)**. Arrowheads show that FOXP2 and CASK also co-localize in Area X, where DAPI is effectively taken up by medium spiny neurons.
